# The Disorders of Digestion in Infancy and Childhood

**Published:** 1898-03

**Authors:** 


**The Disorders of Digestion in Infancy and Childhood.** By W. Soltau Fenwick, M.D. Pp. xiv., 377. London: H. K. Lewis. 1897.

Dr. Fenwick's contribution to the literature of the intestinal disorders of infancy and childhood is a scholarly and useful treatise, and gives evidence of careful observation in the hospital and practical conclusions drawn from a wide field.

Commencing with chapters on the anatomy and physiology of the stomach, the normal diet of children, and simple dyspepsia, he gives an excellent review of what he calls acute gastro-intestinal catarrh, including under this heading several disorders that are usually given a separate description. This disease he divides into two classes—the simple, or that caused by local irritation and the general, or toxæmic. This subdivision appears to us a very useful one, but it is not always so easy to determine clinically which variety is present. In the treatment of this disorder he calls attention to the great difference there is between theory and practice in the use of intestinal antiseptics, and points out that many of these drugs do not react in the same way in the bowel as in the test tube. Notwithstanding, he is evidently a firm believer in their use, and recommends several which experience has taught him are of value.

In the chronic form of the disease the almost constant absence of HCl in the gastric secretion is remarked on, and he points out what a retarding effect this disorder frequently has on the child's subsequent mental and physical growth.

The chapter on "Weak Digestion" is a good one and full of practical suggestions; but we are inclined to think that masturbation as a cause of weak digestion might have been left out.

A chapter is devoted to ulceration of the stomach, and medical literature has been ransacked for cases. That it does occur under the fourteenth year is known; but, as Dr. Fenwick notes, Rokitansky says he has never seen a case.

The remaining chapters deal with the rare intestinal diseases of children.

The book is a very useful one, full of information on the pathology and treatment of the subject of which it treats; but the style is a little laboured, and the book requires close attention in reading.

